# Long-term evolution of human seasonal influenza virus A(H3N2) is associated with an increase in polymerase complex activity

**DOI:** 10.1093/ve/veae030

**Published:** 2024-05-04

**Authors:** René M Vigeveno, Alvin X Han, Robert P de Vries, Edyth Parker, Karen de Haan, Sarah van Leeuwen, Katina D Hulme, Adam S Lauring, Aartjan J W te Velthuis, Geert-Jan Boons, Ron A M Fouchier, Colin A Russell, Menno D de Jong, Dirk Eggink

**Affiliations:** Department of Medical Microbiology, Amsterdam UMC, Amsterdam, The Netherlands; Department of Medical Microbiology, Amsterdam UMC, Amsterdam, The Netherlands; Department of Chemical Biology and Drug Discovery, Utrecht Institute for Pharmaceutical Sciences, Utrecht University, Utrecht, The Netherlands; Department of Medical Microbiology, Amsterdam UMC, Amsterdam, The Netherlands; Department of Medical Microbiology, Amsterdam UMC, Amsterdam, The Netherlands; Department of Medical Microbiology, Amsterdam UMC, Amsterdam, The Netherlands; Department of Medical Microbiology, Amsterdam UMC, Amsterdam, The Netherlands; Department of Microbiology and Immunology and Division of Infectious Diseases, Department of Internal Medicine, University of Michigan, 1150 W. Medical Center Dr., Ann Arbor, MI 48109, USA; Lewis Thomas Laboratory, Department of Molecular Biology, Princeton University, Washington Road, Princeton, NJ 08544, USA; Department of Chemical Biology and Drug Discovery, Utrecht Institute for Pharmaceutical Sciences, Utrecht University, Utrecht, The Netherlands; Complex Carbohydrate Research Center, University of Georgia, 315 Riverbend Road, Athens, GA 30602, USA; Bijvoet Center for Biomolecular Research, Utrecht University, Padualaan 8, Utrecht 3584 CH, The Netherlands; Department of Chemistry, University of Georgia, 315 Riverbend Rd, Athens, GA 30602, USA; Department of Viroscience, Erasmus MC, Dr. Molewaterplein 50, Rotterdam 3015 GE, The Netherlands; Department of Medical Microbiology, Amsterdam UMC, Amsterdam, The Netherlands; Department of Medical Microbiology, Amsterdam UMC, Amsterdam, The Netherlands; Department of Medical Microbiology, Amsterdam UMC, Amsterdam, The Netherlands; Center for Infectious Disease Control, National Institute for Public Health and the Environment (RIVM), Antonie van Leeuwenhoeklaan 9, Bilthoven 3721 MA, The Netherlands

**Keywords:** Influenza virus, H3N2, evolution, polymerase complex activity, receptor specificity, antigenic drift

## Abstract

Since the influenza pandemic in 1968, influenza A(H3N2) viruses have become endemic. In this state, H3N2 viruses continuously evolve to overcome immune pressure as a result of prior infection or vaccination, as is evident from the accumulation of mutations in the surface glycoproteins hemagglutinin (HA) and neuraminidase (NA). However, phylogenetic studies have also demonstrated ongoing evolution in the influenza A(H3N2) virus RNA polymerase complex genes. The RNA polymerase complex of seasonal influenza A(H3N2) viruses produces mRNA for viral protein synthesis and replicates the negative sense viral RNA genome (vRNA) through a positive sense complementary RNA intermediate (cRNA). Presently, the consequences and selection pressures driving the evolution of the polymerase complex remain largely unknown. Here, we characterize the RNA polymerase complex of seasonal influenza A(H3N2) viruses representative of nearly 50 years of influenza A(H3N2) virus evolution. The H3N2 polymerase complex is a reassortment of human and avian influenza virus genes. We show that since 1968, influenza A(H3N2) viruses have increased the transcriptional activity of the polymerase complex while retaining a close balance between mRNA, vRNA, and cRNA levels. Interestingly, the increased polymerase complex activity did not result in increased replicative ability on differentiated human airway epithelial (HAE) cells. We hypothesize that the evolutionary increase in polymerase complex activity of influenza A(H3N2) viruses may compensate for the reduced HA receptor binding and avidity that is the result of the antigenic evolution of influenza A(H3N2) viruses.

## Introduction

Seasonal influenza viruses infect 5–15 per cent of the global population yearly, resulting in substantial morbidity and an estimated 290,000–645,000 deaths annually (Iuliano et al. 2018). Currently, subtypes A(H3N2) and A(H1N1pdm09) are circulating with influenza B viruses among humans. In order to establish continued circulation in the human population after their pandemic introduction, seasonal influenza viruses must escape immunity build up in the human population as a result of prior infections or vaccination. Immune escape of seasonal influenza viruses occurs via genetic drift. Genetic drift leads to the gradual accumulation of mutations in the influenza virus genome over time due to the intrinsic low fidelity of the influenza virus polymerase complex (Bouvier and Palese 2008; Taubenberger and Kash 2010) and selective pressure including host immunity. Seasonal influenza viruses escape from humoral immunity through changes of the antigenic properties of the hemagglutinin (HA) and neuraminidase (NA) surface glycoproteins. Amino-acid substitutions in HA or NA result in either a change in antibody epitopes or the addition of N-glycan-moieties, which may shield epitopes against antibody binding. In immunized host populations, such mutated viruses have an advantage over viruses without these mutations and are selected (Altman et al., 2019; Smith et al. 2004; Sandbulte et al. 2011; York, Stevens, and Alymova 2019).

Although much is known about the evolution of the influenza virus surface glycoproteins in relation to antigenic properties, vaccine strain selection, receptor binding, and receptor specificity, less is known on the evolution of the influenza virus RNA polymerase complex. The influenza virus RNA polymerase complex, which consists of the polymerase basic 1 (PB1), polymerase basic 2 (PB2), and polymerase acidic (PA) subunits, forms a hetero-trimer which, together with nucleoprotein (NP) and viral RNA (vRNA), constitute the viral ribonucleoprotein (vRNP) complex. The vRNP complex serves to replicate the eight negative sense vRNA genome segments via a positive-sense complementary RNA intermediate (cRNA), and generates mRNA for viral protein synthesis (Bouvier and Palese 2008; Taubenberger and Kash 2010). Previous phylogenetic studies have demonstrated continued evolution in all eight gene segments of the influenza A(H3N2) virus genome since 1968 (Nelson et al. 2006; Bragstad, Nielsen, and Fomsgaard 2008; Westgeest et al. 2014). Although the HA and NA gene segments demonstrated the highest rates of nucleotide substitutions over time, the RNA polymerase complex gene segments PB2, PB1, PA together with NP as well as the M and NS gene segments have also evolved significantly since the 1968 influenza A(H3N2) pandemic (Westgeest et al. 2014). Surprisingly, little is known about the functional implications of RNA polymerase complex evolution in seasonal influenza viruses. Furthermore, whereas for HA and NA the selection pressures driving antigenic evolution are well established, the selection pressures driving RNA polymerase complex evolution are less well understood.

Most studies investigating the influenza virus polymerase complex function have focused on host-adaptation of avian influenza viruses to mammalian hosts in relation to transmission and virulence (Gabriel et al. 2007; Manz et al. 2016). Numerous substitutions in the polymerase complex subunits have been identified as virulence factors, such as PB2 E627K (Subbarao, London, and Murphy 1993; Welkers et al. 2019), PB2 D701N (Czudai-Matwich et al. 2014), PB2 271A (Bussey et al. 2010), and PA 97I (Song et al. 2009) as well as mutations in NP such as NP 319K (Gabriel et al. 2005). These mutations affect RNA polymerase complex activity of avian influenza A viruses in mammalian hosts, thereby affecting replication and virulence. Few studies have investigated RNA polymerase complex function of seasonal influenza viruses, most of which investigated the effect of reassorted polymerase complexes between seasonal influenza viruses (Lam, Ngai, and Chan 2011; Phipps et al. 2017), or in respect to specific polymerase complex functions such as RNA polymerase complex fidelity (Cheung et al. 2014), temperature sensitivity (Da Costa et al. 2015), or the role of specific mutations such as PB2 K526R and E627K (Song et al. 2014). To date, few studies have systematically evaluated the phenotypical evolution of seasonal influenza virus RNA polymerase complexes.

The aim of our study was to characterize the genetic and functional evolution of the influenza A(H3N2) virus RNA polymerase complex. We selected eight viruses, spanning approximately 50 years of seasonal influenza A(H3N2) virus evolution, from 1968 to 2017 and quantified their RNA polymerase complex activity, investigated the balance between replication and transcription and assessed the role of the RNA polymerase complex in infection experiments in MDCK and A549 cells as well as a human airway epithelium (HAE) model.

## Material and methods

### Phylogenetics seasonal influenza A(H3N2) viruses

All human influenza A(H3N2) virus isolates collected from 1968 until 2018 with full genomes were downloaded from the GISAID EpiFlu database (https://www.gisaid.org). A total of 18,305 viral sequences remained after removing duplicates and entries with low quality sequence data (i.e. >90 per cent of full gene segment length and <1 per cent ambiguous nucleotide residues in all gene segments). To reduce the number of sequences used for phylogenetic reconstruction, we randomly down sampled the sequence collection (*n* = 1,327). For each set, we ensured that an even distribution of sequences was sampled annually between 1968 and 2017 and that all six continental regions (i.e. Africa, Asia, Europe North America, South America, and Oceania) were represented. We aligned the sequences of individual gene segments using MAFFT (Katoh and Standley 2013) and manually edited them to obtain codon alignments. TempEst (Rambaut et al. 2016) was used to identify and discard any sequences that violated the molecular clock assumption as a result of sequencing errors or misdating. Finally, the remaining dataset was used to reconstruct gene phylogenies with the GTR + I + G substitution model in IQtree (Nguyen et al. 2015).

### Cell culture

Human A549 (ATCC CLL-185), HEK293T (ATCC CRL-3216), and HEK293T RIG-I deficient cells (Vigeveno et al. 2020) were cultured in Dulbecco’s Modified Eagle’s Medium (DMEM; Gibco) supplemented with 10 per cent fetal calf serum (FCS; Sigma), 1 per cent non-essential amino acids (NEAA; Gibco), 100 IU/ml penicillin, and 100 IU/ml streptomycin (Pen/Strep; Gibco) incubated at 37°C/5 per cent CO_2_.

MDCK London-Luc 9.1 cells (Hossain et al. 2010) were cultured in Eagle’s minimal essential medium (EMEM, Lonza), supplemented with 10 per cent fetal calf serem (FCS; Sigma), 1 per cent non-essential amino acids (NEAA; Gibco), 2 nM L-glutamine (Gibco), 500 µg/ml G418, and 10 mM HEPES (Lonza).

Primary human airway epithelial cells (HAE) were isolated from patients (>18 years old) who underwent bronchoscopy and/or surgical lung resection in their diagnostic pathway for any pulmonary disease. This was carried out in accordance with local regulations from the Amsterdam University Medical Center. Healthy epithelial cells from the tissue samples were isolated and cultured according to Fulcher’s protocol (Fulcher and Randell 2013). For this study, cells from two randomly selected donors were used for two HAE viral replication experiments, both in triplicate. Primary nasal airway epithelial cells were maintained in PneumaCult-ExPlus medium (StemCell) in T75 flasks coated with type-I collagen (VitroCol, 5007-20ML, Advanced BioMatrix). Upon reaching 80–90 per cent confluency, cells were trypsinized and resuspended in PneumaCult-ExPlus medium to 0.4 µM pore-size transwell inserts (3740, Corning) which were coated with type-IV collagen (C7521-10MG, Sigma). When confluency was reached, apical media were removed and basolateral media were replaced with in PneumaCult-Ali medium (StemCell) to initiate differentiation at the air–liquid interface over 6 weeks.

### Polymerase activity/mini genome assay

The open reading frames (coding region) of the PB2, PB1, PA, and NP genes were cloned into the pPPI4 expression vector (Binley et al. 2000) using Gibson assembly (New England Biolabs). A plasmid coding for a model viral RNA (vRNA), consisting of the firefly luciferase (FF) open reading frame flanked by the non-coding regions of segment 8 of influenza A virus under the control of a human RNA polymerase I promoter (PolI), was used to provide the template for mini genome assays (Manz et al. 2016). Production and translation of mRNAs from this FF vRNA depend on transcription of the template vRNA by the influenza virus RNA polymerase complex. Therefore, the FF activity is a measure of the amount of mRNA produced by the polymerase complex activity. Transfection of pRL (Promega), a plasmid containing the *Renilla* luciferase gene under a cytomegalovirus promotor which results in constitutive expression in avian and mammalian cells independent from the influenza polymerase complex, served as an internal control to normalize variation in transfection efficiency and sample processing. Human HEK293T or A549 cells were seeded one day prior to the experiment in 96 wells plates. 25 ng of the FF reporter plasmid, 50 ng of each of the plasmids encoding PB2, PB1, and PA, and 100 ng of NP and 2 ng of the pRL expression plasmid in 50 µl Opti-MEM (Gibco, ThermoFisher) were mixed with 50 µl Opti-MEM containing either Lipofectamine2000 (Invitrogen, ThermoFisher) for HEK293T cells or Trans-IT-X2 (Mirius) for A549 cells in a 1:3 ratio and incubated for 20–30 min at room temperature. 20 µl of the transfection mixture was added to each well. Each transfection was performed in quadruplicates in at least three independent experiments. At 4, 8, 12, 16, or 24 h after transfection, luminescence was measured using the Dual-Luciferase Reporter Assay System (Promega) using a GloMax luminometer according to the manufacturer’s instructions (Turner BioSystems).

### Quantitative PCR luciferase viral, complementary, and messenger RNA

Transfection of cells was performed as described above for the minigenome experiments. Briefly, 25 ng of the FF reporter plasmid, 50 ng of each of the plasmids encoding PB2, PB1, and PA, and 100 ng of NP and 2ng of the pRL expression plasmid in 50 µl Opti-MEM (Gibco, ThermoFisher) were mixed with 50 µl Opti-MEM containing Lipofectamine2000 (Invitrogen, ThermoFisher). HEK293T cells, cultured in 24 well format, were transfected with 100 µl transfection mixture and incubated for 24 h after which media was aspirated and cells were lysed in 500 µl Trizol (ThermoFisher) and stored at −80°C until RNA isolation. Transfections were performed in triplicate. For each transfection, a separate cDNA synthesis and qPCR was performed in duplicate. RNA isolation from Trizol was performed according to manufacturer’s protocol. To ensure complete removal of any residual DNA (genomic/plasmid), the isolated RNA was treated with 100 units of Turbo DNase (ThermoFisher) for 2 h and RNA was isolated a second time using Trizol.

For cDNA synthesis, 1 µg of purified total RNA was used to which 1 µl oligo(dT)20 ([50 µM]; ThermoFisher) or 1 µl gene-specific primer (2 µM) was added and 1 µl dNTP mix ([10 mM]; ThermoFisher) in a total volume of 13 µl. Oligo(dT) (Phipps et al. 2017) was used to generate mRNA, gene-specific primers for luciferase vRNA (5ʹ-AGTAGAAACAAGGGTGTTTTTTATCA-3ʹ) and cRNA (5ʹ-TATGGGCATTTCGCAGCCTACCGTGGTGTT-3ʹ) (adapted from (Obayashi et al. 2008), (Long et al. 2016)). This mixture was heated to 65°C for 5 min then incubated on ice for at least 1 min. Next, 4 µl first strand buffer (FSB [5x], ThermoFisher), 1 µl DTT ([0.1 m]; ThermoFisher), 1 µl RNaseOUT (ThermoFisher), and 1 µl SuperScript III reverse transcriptase ([200 U/µl]; ThermoFisher) were added to each reaction and incubated at 55°C for 60 min, 75°C for 15 min, and cooled to 4°C. For the real-time quantitative PCR (qPCR), 2 µl of cDNA was used, and 10 µl of KAPA SYBER FAST qPCR master mix for LC480 ([2x], KAPA Biosystems, KK4610], 4 µl PCR grade water, and 2 µl forward primer (2 µM) and 2 µl reverse primer (2 µM) were used for each reaction. For detection of luciferase vRNA, cRNA, and mRNA, luciferase qPCR primers were used (forward: 5ʹ- TATGAACATTTCGCAGCCTACCGTAGTGTT- 3ʹ, reverse: 5ʹ- CCGGAATGATTTGATTGCCA-3ʹ) (Obayashi et al. 2008; Long et al. 2016). For quantification of household gene GAPDH, GAPDH mRNA was detected using qPCR primers (forward: 5ʹ-AAAATCAAGTGGGGCGATGCT-3ʹ, reverse: 5ʹ-GGGCAGAGATGATGACCCTTT-3ʹ) (Wang et al. 2016). Real-time quantitative PCR using KAPA SYBER FAST qPCR mix for LC480 was performed on LightCycler480 (Roche) according to manufacturer’s protocol. Gene expression relative to negative control was calculated using the delta-delta CT method (Livak and Schmittgen 2001) using GAPDH as a household gene.

### Virus isolates

All Influenza A(H3N2) virus isolates (Table 1) were propagated in MDCK cells as previously described (Westgeest et al. 2015).

**Table 1.** Influenza A(H3N2) viruses used to characterize the seasonal A(H3N2) influenza polymerase complex.

Abbreviated	Isolate name	Collection date (year–month–day)	Season	Accession number GISAID
1968	A/Bilthoven/16190/68	1968	1968	EPI_ISL_124885
1972	A/Bilthoven/21793/72	1972	1972	EPI_ISL_110818
1982	A/Netherlands/233/82	1982-4-1	1981-1982	EPI_ISL_115699
1993	A/Netherlands/179/93	1993-2-26	1992-1993	EPI_ISL_111045
2003	A/Netherlands/213/03	2003-4-20	2002-2003	EPI_ISL_84455
2008	A/Netherlands/377/08	2008-9-27	2008-2009	EPI_ISL_110724
2014	A/Netherlands/443/14	2014-11-25	2014-2015	EPI_ISL_170315
2017	A/Netherlands/499/17	2017-1-11	2016-2017	EPI_ISL_242387

### Recombinant viruses

The eight gene segments of influenza A(H3N2) viruses were amplified from RNA by one-step Reverse Transcriptase Polymerase Chain Reaction (ThermoFisher) using specific primers and were cloned in the bidirectional reverse genetics plasmid pHW2000 as described before (Hoffmann et al. 2000; de Wit et al. 2004). Recombinant viruses were rescued by reverse genetics upon transfection of HEK293T cells as previously described (de Wit et al. 2004). Virus stocks were propagated in MDCK cells and titrated on MDCK cells as described below.

### Titrations

MDCK cells were inoculated with ten-fold serial dilutions of virus stocks or supernatant of HAE inoculated cells. The cells were washed with phosphate-buffered saline (PBS) 1 h after inoculation and cultured in infection medium consisting of EMEM supplemented with 100 IU/ml penicillin, 100 IU/ml streptomycin, 2 mM L-glutamine (Gibco), 0.15 per cent sodium bicarbonate (Gibco), 10 mM HEPES (Gibco), and 1 per cent NEAA (Gibco), incubated at 37°C/5 per cent CO_2_. Three days after inoculation, supernatants of cell cultures were tested for agglutinating activity using turkey red blood cells (tRBCs) as an indicator of virus replication. Infectious virus titers were calculated by the method of Reed and Muench (Reed and Muench 1938).

### Polymerase complex activity in virus-inoculated MDCK reporter cells

MDCK-London-Luc reporter cells (International Reagent Resource, FR-1355) were seeded in 96 wells plates one day prior to influenza A(H3N2) virus inoculation. Cells were aspirated, rinsed with PBS, and inoculated with a MOI of 0.1 in infection medium consisting of EMEM supplemented with 100 IU/ml penicillin, 100 IU/ml streptomycin, 2 mM L-glutamine (Gibco), 0.15 per cent sodium bicarbonate (Gibco), 10 mM HEPES (Gibco), and 1 per cent NEAA (Gibco), incubated at 37°C/5 per cent CO_2_. Virus inoculation was performed in eight replicates in three independent experiments. Then, 24 h post inoculation, cells were aspirated and lysed using passive lysis buffer (Promega). *Renilla* luminescence was measured using the *Renilla* luciferase detection agent (Dual-Luciferase Reporter Assay System, [Promega]) using a GloMax luminometer according to the manufacturer’s instructions (Turner BioSystems).

### MDCK, A549, and Human airway epithelium viral replication

MDCK and A549 cells were inoculated in triplicate with 0.001 multiplicity of infection (MOI), and samples were collected up to 72 h after inoculation. Differentiated nasal human airway epithelial cells (HAE) were inoculated with influenza A(H3N2) viruses by adding 10^4^ TCID_50_ in HBSS to the apical side of the transwell insert. Inoculation was performed in triplicate at 33°C in two independent experiments. Following inoculation cells were incubated for 2 h, after which the inoculum was removed and the apical side was washed twice with HBSS. At 6, 12, 24, 36, 48, and 72 h after inoculation, the apical side of the epithelial cells was sampled by washing with 200 µl HBSS for 20 min. Samples were stored at −80°C until titration.

### Glycan micro-array

Glycan micro-array analysis of influenza A (H3N2) virus isolates was performed as described previously (Stevens et al. 2010; Peng et al. 2017; Eggink et al. 2020; Broszeit et al. 2021). Briefly, glycan compounds were printed on glass slides in six replicates which were subsequently incubated with control lectins and influenza A(H3N2) virus isolates. Lectins were applied precomplexed with Streptavidin-Alexa-555 at a 4:1 ratio. Viruses were incubated in the presence of oseltamivir; thereafter, glass slides were rinsed and incubated first with influenza virus HA stem-specific antibodies and subsequently with a secondary goat anti-human Alexa-647 antibody. The stained slides were scanned using an Innopsys Innoscan 710 microarray scanner at the appropriate excitation wavelength. Images were analyzed with Mapix software (version 8.1.0 Innopsys). The average fluorescence intensity and SD were measured for each compound after exclusion of the highest and lowest intensities from the spot replicates (*n* = 4).

### Statistical analysis

Mini-genome assay polymerase complex activities of influenza A(H3N2) viruses were compared using the students *t*-test (Prism 8.0.2, Graphpad). A *P*-value <0.05 was considered statistically significant. In the figures, we indicated significance as * *P* < 0.05; ** *P* < 0.005; *** *P* <0.0005, ns not significant.

## Results

### Phylogenetics and amino-acid substitutions in the polymerase complex genes of influenza A(H3N2) viruses demonstrate ongoing evolution since 1968

**Figure 1.**
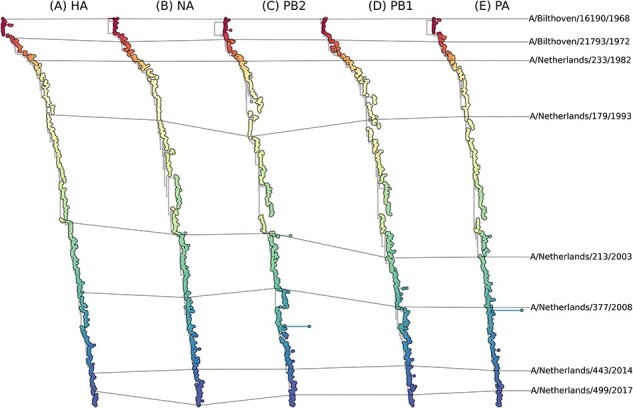
**Phylogenetic analysis demonstrates ongoing genetic evolution of human influenza A(H3N2) virus HA, NA, PB2, PB1, and PA gene segments between 1968 and 2017**. Phylogenetic trees of influenza A(H3N2) gene segments HA (A) and NA (B) as well as the polymerase complex gene segments PB2 (C), PB1 (D), and PA (E) demonstrate ongoing genetic evolution from 1968 onward. Trees are color-coded to antigenic evolution based on antigenic mapping (Smith et al. 2004; Westgeest et al. 2014) of H3N2 HA. Influenza A(H3N2) viruses used for subsequent phenotypic characterization are annotated in each phylogenetic tree.

To investigate the genetic evolution of the three influenza A(H3N2) virus RNA polymerase complex encoding genes in relation to the evolution of HA and NA encoding genes, we reconstructed maximum-likelihood phylogenetic trees for the HA, NA, PB2, PB1, PA, and NP encoding gene segments of influenza A(H3N2) virus isolates collected globally between 1968 and 2017. After rooting each tree by maximizing the correlation between time and genetic divergence, each tree displayed a similar ‘ladder-like’ topology (Fig. 1, supplemental Fig. S1), which is characteristic of gradual genetic evolution as previously described (Westgeest et al. 2014). The evolutionary rate as measured in the genetic distance to the 1968 pandemic virus, of the HA and NA gene segments (Fig. 1A,B), exceeded that of the PB2, PB1, and PA gene segments (Fig. 1C–E) and the NP gene segment (supplemental Fig. S1B), showing higher nucleotide substitution rates for HA and NA compared to the polymerase complex genes in line with earlier studies (Westgeest et al. 2014).

**Figure 2.**
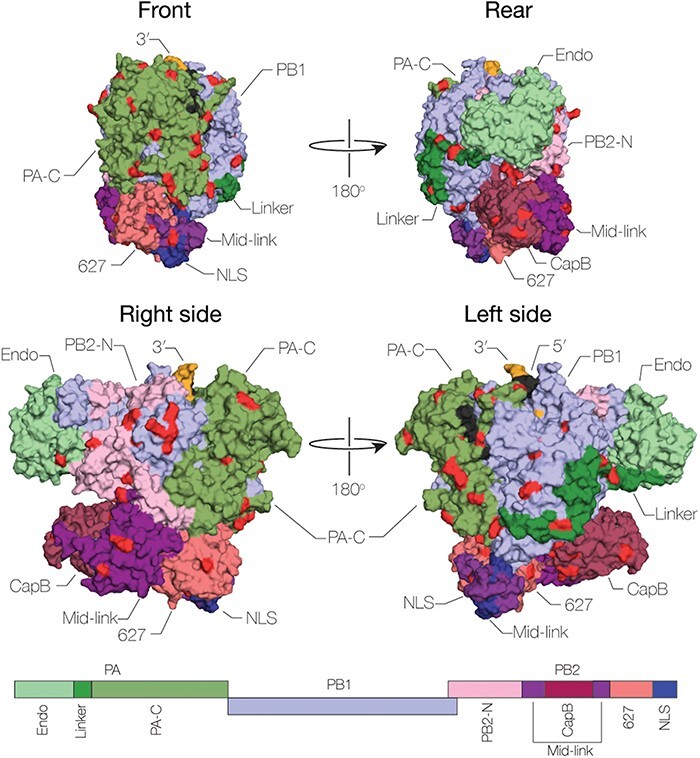
**Surface presentation of substitutions of the transcription initiation structure of the influenza A virus H3N2 RNA polymerase**. The substitutions identified in this study were mapped on the structure of influenza virus A(H3N2) polymerase (DB 6RR7) in PyMol and highlighted in red. The structural domains of the RNA polymerase are indicated with the follow abbreviations: PA endonuclease (Endo), PA linker domain (Linker), PB2 N-terminal domain (PB2-N), PB2 cap binding domain (CapB), PB2 nuclear localization signal (NLS), PB2 627-domain (627), and the PA C-terminal domain (PA-C). Three different orientations of the complex are presented with indicated angles.

We next selected eight viruses (Table 1) that are representative of the genetic evolution of influenza A/H3N2 viruses for subsequent functional assays. Interestingly, large portions of each of the gene segments do not exhibit any changes in amino-acid composition over time, suggesting that these represent conserved regions that are linked to essential enzymatic functions or interactions with accessory host-factors of the influenza A(H3N2) virus polymerase complex. However, some amino acid changes do occur. Figure 2 and supplemental Table S1 show all amino-acid changes (*n* = 111 in total) in the selected influenza A(H3N2) viruses compared to the 1968 reference influenza A(H3N2) virus. The amino-acid changes were classified as ‘fixed’ (*n* = 85) when a given amino-acid change was found in all subsequent later viruses after their introduction, ‘unique’ (*n* = 14) when they occur once in one of the eight viruses, or ‘variable’ (*n* = 12) when multiple amino-acid changes occur at one position over time. The majority of these amino acid changes have not yet been described.

### Polymerase complex activity of influenza A(H3N2) viruses increased from 1968 to 2017

To study the impact of the above amino acid changes on the phenotype evolution of the influenza A(H3N2) polymerase complex, we first quantified the polymerase complex activity of the eight selected influenza A(H3N2) viruses isolated in 1968, 1972, 1982, 1993, 2003, 2014, and 2017 (Table 1) using mini-genome assays. Briefly, the coding sequences of the PB2, PB1, PA, and NP segments were cloned into expression vectors and the resulting plasmids transfected into cells together with a FF luciferase reporter plasmid consisting of a PolI promoter driving expression of the FF open reading frame flanked by the non-coding regions of influenza A virus segment 8. The measured luciferase signal reflects the influenza A virus RNA polymerase complex transcriptional activity. The polymerase complex activity of these viruses was first measured in human HEK293T cells 24 h after transfection at 37°C (Fig. 3A). Seasonal influenza A(H3N2) viruses exhibited high polymerase complex activity in human cells. To visualize differences in activity between the viruses, the RNA polymerase complex activity was normalized to the activity of the 1968 influenza A(H3N2) virus (Table 2). The polymerase complex activity of influenza A(H3N2) viruses increased steadily from 1968 to 2008 with the exception of the 2003 influenza A(H3N2) polymerase complex. After 2008, the polymerase complex activity decreased slightly. The most active RNA polymerase complex was that of the 2008 influenza A(H3N2) virus, which was approximately 5.9-fold (*P* < 0.0005) more active compared to the 1968 influenza A(H3N2) virus polymerase complex.

**Figure 3.**
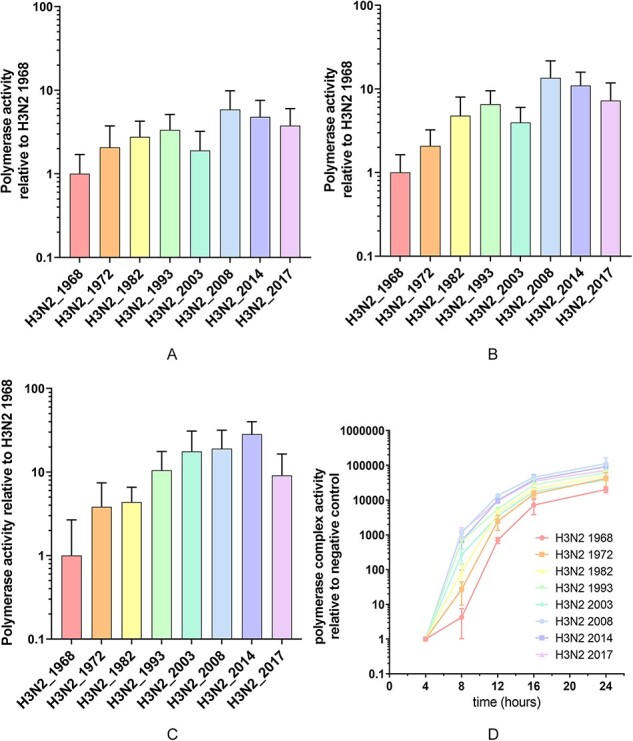
**The polymerase complex activity of human influenza A(H3N2) viruses increases over time**. Polymerase complex activity of eight influenza A(H3N2) viruses from 1968 to 2017 as measured in minigenome assays in (A) HEK293T cells incubated at 37°C, (B) HEK293T cells incubated at 33°C, (C) A549 cells incubated at 37°C. (D) Polymerase complex activity as measured in mini-genome assays at 4, 8, 12, 16, and 24 h after transfection in HEK293T cells incubated at 37°C. (E) Polymerase complex activity of influenza A(H3N2) viruses from 1968 and 2017 and reassortants thereof in HEK293T cells incubated at 37°C. (F) Polymerase complex activity of eight influenza A(H3N2) viruses from 1968 to 2017 in virus inoculated MDCK reporter cells. (G) Quantitative PCR for luciferase vRNA, cRNA, and mRNA in HEK293T cells transfected with the polymerase complex of eight influenza A(H3N2) viruses from 1968 to 2017. For mini-genome assays, data were normalized to the polymerase complex activity of the influenza A(H3N2) virus from 1968. For MDCK reporter assay, data were normalized to negative control. Bar graphs and time-series show means with SD from three independent experiments each done in quadruplicate. Expression levels of vRNA, cRNA, and mRNA were calculated relative to negative control using GAPDH as a household gene. Bar graphs show means with SD from three independent experiments.

**Figure 3.**
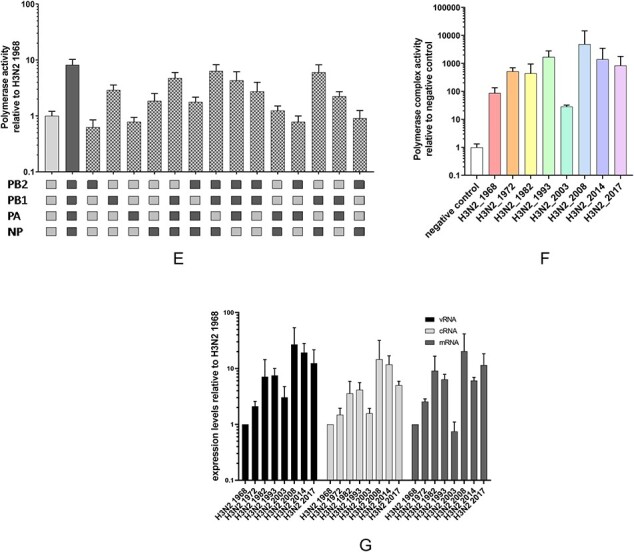
continued

**Table 2.** Polymerase complex activity of seasonal influenza A(H3N2) viruses in HEK293T and A549 cells relative to the 1968 influenza A(H3N2) virus polymerase complex activity.

Influenza A(H3N2) viruspolymerase complex	HEK293T cells 37°C, 24 h	HEK293T cells 33°C, 24 h	A549 cells 37°C, 24 h	HEK293T cells 37°C, 8 h
1968	1	1	1	1
1972	2.1-fold *	2.1-fold **	3.8-fold *	6.8-fold **
1982	2.8-fold ***	4.8-fold ***	4.4--fold ***	20.0-fold ***
1993	3.4-fold ***	6.6-fold ***	10.4-fold ***	144.1-fold ***
2003	1.9-fold *	4.0-fold ***	17.7-fold ***	61.8-fold ***
2008	5.9-fold ***	13.5-fold ***	19-fold ***	250.1-fold ***
2014	4.8-fold ***	11.0-fold ***	28.4-fold ***	167.5-fold **
2017	3.8-fold ***	7.2-fold ***	9.1-fold **	260.6-fold ***

Polymerase complex activity was measured in mini-genome assays at 8 or 24 h after transfection, incubated at 33°C or 37°C. Data were normalized to the 1968 influenza A(H3N2) virus polymerase complex activity. Fold increases in polymerase complex activity relative to the activity of the 1968 virus polymerase complex were compared using a two-tailed unpaired *t* test.

* *P* < 0.05, ** *P* < 0.005, *** *P* <0.0005.

Next, we wanted to investigate the effects of temperature on the polymerase complex activity. We therefore performed the same experiment in HEK293T cells at 33°C, resembling the temperature of the human upper respiratory tract (Fig. 3B). The pattern of polymerase complex activity for the eight influenza A(H3N2) viruses at 33°C was similar to that at 37°C, increasing from 1968 to 2017, although the differences in polymerase complex activity between the eight influenza A(H3N2) viruses were more pronounced at 33°C (Table 2). The most active polymerase complex was that of the 2008 influenza A(H3N2) virus, which was approximately 13.5-fold (*P* < 0.0005) more active than the 1968 influenza A(H3N2) virus polymerase complex at 33°C.

Since HEK293T cells are derived from human embryonic kidney cells, we also wanted to test the polymerase complex activity in a more representative cell line of pulmonary origin. We therefore quantified the polymerase complex activity in A549 cells, which are derived from a human lung cell carcinoma. In A549 cells at 37°C (Fig. 3C, Table 2), the pattern of increased polymerase complex activity over time for the eight influenza A(H3N2) viruses is comparable to HEK293T cells, with an increase in polymerase complex activity from 1968 onward, with the exception of the 2003 polymerase complex. Remarkably, whereas the activity of the 2003 influenza A(H3N2) polymerase complex was approximately 1.9-fold (*P* < 0.05) higher compared to the 1968 virus in HEK293T cells, in A549 cells, the activity of the 2003 polymerase complex was approximately 17.7-fold (*P* < 0.0005) higher compared to the 1968 polymerase complex activity in A549 cells. In A549 cells, the most active polymerase complex was that of the 2014 virus, which was 28.3-fold (*P* < 0.0005) more active compared to the 1968 influenza A(H3N2) polymerase complex.

To investigate the kinetics of polymerase activity, we measured polymerase complex activity at 4, 8, 12, and 16 h after transfection in addition to the 24-h incubation time (Fig. 3D). The earlier time-points after transfection show increased differences in polymerase complex activity between viruses. For example, whereas for the 2014 influenza A(H3N2) virus the polymerase complex activity is approximately five-fold higher than that of the 1968 influenza A(H3N2) polymerase complex activity at 24 h, at 8 h the activity of the 2017 influenza A(H3N2) polymerase complex is approximately 260-fold higher compared to the activity of the 1968 polymerase complex.

To identify which subunit(s) of the RNA polymerase complex contributed most to the increase of polymerase complex activity, we reassorted the polymerase complex subunits from the 1968 influenza A(H3N2) virus with those of the 2017 influenza A(H3N2) virus (Fig. 3E). The relative low RNA polymerase complex activity of the 1968 influenza A(H3N2) virus was markedly increased when either the PB1 or NP gene segment of the 2017 influenza A(H3N2) virus were combined with the remaining gene segments of the 1968 influenza A(H3N2) virus. When both the PB1 and NP gene segments of the 2017 virus were combined with the PB2 and PA gene segments of the 1968 virus, the polymerase complex activity was similar to that of the full 2017 virus. Interestingly, whereas the PA gene segment of the 1982 virus combined with the PB2, PB1, and NP of the 1968 virus did not increase the polymerase complex activity, combining the PA gene segment and the PB1 gene segment of the 1968 virus with the PB2 and NP from the 2017 virus resulted in a marked increase of polymerase complex activity, although not to the same level as addition of 2017 PB1 and NP. To confirm our results, we reassorted the polymerase complex subunits of the 1968 virus with those of the 1972 and the 1982 influenza A(H3N2) viruses and tested their polymerase complex activity. This analysis demonstrated that the PB1 and NP consistently contributed most to the increase in polymerase complex activity from 1968 to 2017 (supplemental Fig. S2), although the effect was more pronounced when we reassorted the 1968 with the 2017 polymerase complex.

To test whether the polymerase complex activity as measured in mini-genome assays resembles polymerase complex activity in virus infected cells, we inoculated MDCK-London-Luc reporter cells with the eight influenza A(H3N2) viruses from 1968 to 2017 (Table 1). MDCK-London-Luc cells are a reporter cell line that stably express a vRNA template encoding the *Renilla* luciferase. This vRNA is transcribed by the influenza virus polymerase complex upon inoculation and *Renilla* luciferase activity can subsequently be used as a measure for polymerase complex activity in the inoculated cells. We inoculated MDCK reporter cells at a high MOI 0.1 and incubated them for 24 h to capture the early stages after infection (Fig. 3F). The RNA polymerase complex activity of the eight selected influenza A(H3N2) viruses from 1968 to 2017 in virus inoculated cells was similar to that of transfected polymerase complexes in mini-genome assays. However, the 2003 influenza A(H3N2) virus polymerase complex exhibited lower polymerase complex activity compared to the 1993 and 2008 influenza A(H3N2) viruses in the inoculated cells, in line with our other results.

### Balance between replication and transcription is conserved over time

During infection, the influenza virus polymerase complex transcribes a vRNA template into mRNA for protein synthesis and a cRNA to generate negative-sense vRNA for virus replication. Although not fully understood, a balanced replication/transcription process is believed to be important for virus replication and fitness and thereby possibly adaptation or virulence. We next investigated whether the balance between influenza A(H3N2) virus replication and transcription changed during its evolution from 1968 onward. To test this, we transfected plasmids encoding the polymerase complex genes together with the luciferase vRNA reporter plasmid in HEK293T cells for each of the eight influenza A(H3N2) viruses from 1968 to 2017. After 24 h, total RNA was isolated, cDNA synthesized using strand specific primers for luciferase vRNA and cRNA and oligo(dT)20 for mRNA (Obayashi et al. 2008; Long et al. 2016), and qPCR performed (Fig. 3G). For each of the RNA species, the expression levels of the eight influenza A(H3N2) viruses showed a pattern similar to that of the luciferase signal measured in the mini-genome assays: increasing from 1968 onward. The balance between cRNA, vRNA, and mRNA expression levels was well conserved, with the exception of the 2003 influenza A(H3N2) polymerase complex which produced low levels of mRNA but relatively higher levels of cRNA and vRNA. Also, the 2014 influenza A(H3N2) polymerase complex produced slightly lower mRNA levels than expected based on the mini-genome assay data. However, mRNA expression levels of influenza A(H3N2) viruses measured in the qPCR experiments corresponded well with both activity as measured in the mini-genome assay polymerase activity and the virus-inoculated MDCK reporter cell activity.

### Titers of influenza A(H3N2) viruses on A549 cells correlates with polymerase complex activity, but titers on MDCK cells do not

**Figure 4.**
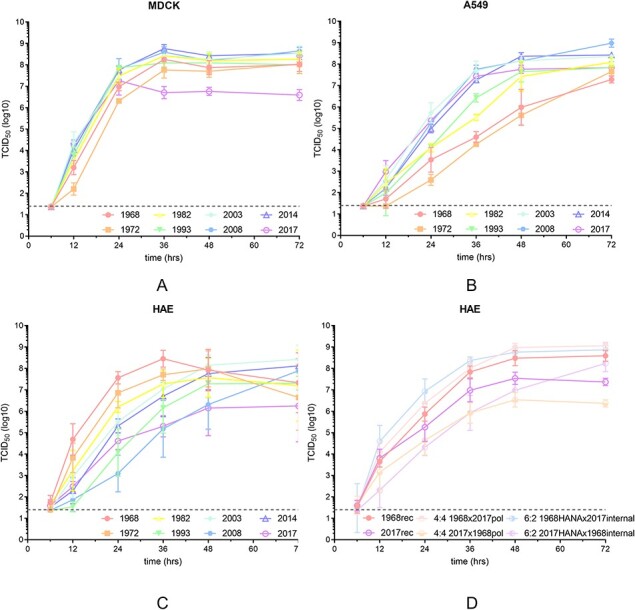
**Replication of human influenza A(H3N2) viruses on A549 does, but on HAE does not correlate with polymerase complex activity**. H3N2 virus replication of influenza A(H3N2) clinical isolates was investigated on conventional cell lines MDCK (A), A549 (B), and human differentiated primary airway epithelial cells (C). MDCK and A549 cells were inoculated with 0.001 MOI and HAE cells were inoculated with 10^4^ TCID50 with each of the influenza A(H3N2) viruses at the apical side of the transwell insert following 6 weeks of HAE differentiation. Samples were collected up to 72 h after inoculation for titration on MDCK cells. (D) Virus replication on HAE of reassorted recombinant 1968 and 2017 viruses containing the four polymerase genes or the HA and NA genes of the contrasting strain.

To test whether the increase in polymerase complex activity of seasonal influenza A(H3N2) viruses from 1968 to 2017 also resulted in increased virus replication, MDCK and A549 cells were inoculated with 0.001 MOI of all eight clinical isolates and samples were collected up to 72 h after inoculation. Figure 4 shows that the titers of seasonal influenza A(H3N2) viruses in A549 cells at 37°C generally agreed with the differences observed in the RNA polymerase complex activity as measured in mini-genome assays, with the viruses from 2003 onwards showing the fastest growth kinetics and the 1968 and 1972 viruses showing the slowest growth kinetics. In contrast, these differences were less pronounced on MDCK cells. On MDCK cells, the 1968 and 1972 initially showed minor delays in growth, but eventually reached titers that were within 1 log of all other viruses, except the 2017 A(H3N2) isolate, whose titers were about 2 logs lower than the other viruses.

### Titers of influenza A(H3N2) viruses on HAE do not correlate with polymerase complex activity

To test whether the increase in polymerase complex activity of seasonal influenza A(H3N2) viruses from 1968 to 2017 resulted in increased virus replication in primary human airway epithelium (HAE) cells, HAE were inoculated with the eight clinical isolate influenza A(H3N2) viruses from 1968 to 2017 and samples collected up to 72 h after inoculation. Figure 4C shows that the replication of seasonal influenza A(H3N2) viruses in HAE cells at 37°C was not primarily predicted by polymerase complex activity as measured in mini-genome assays. We observed a reverse-correlation between activity as measured in the mini-genome assay and replication efficiency. Counterintuitively, viruses with low polymerase complex activity in mini-genome assays, such as viruses from 1968, 1972, and 1982, replicated efficiently on HAE and peaked with high titers at 36 h. Viruses with high polymerase complex activity in mini-genome assays, such as viruses from 1993, 2008, and 2014, replicated slower on HAE, reaching titers similar to other influenza A(H3N2) viruses, but peaking later at 48 h. Interestingly, the 2003 influenza A(H3N2) virus, which showed low polymerase complex activity in the mini-genome assay in HEK293T cells but high activity in A549 cells, demonstrated slower replication on HAE comparable to the 2008 and 2014 virus.

To further elucidate the relative contribution of the polymerase complex to viral replication, we used reverse genetics to create several recombinant influenza A(H3N2) viruses, namely a recombinant 1968 wildtype virus, a recombinant 2017 wildtype virus, two recombinant viruses with exchanged polymerase complexes (PB2, PB1, PA, and NP derived from the 1968 virus combined with HA, NA, NS, and MP from the 2017 virus and vice versa) as well as two recombinant viruses where we exchanged HA and NA between the 1968 and 2017 viruses. Figure 4D shows replication kinetics for the recombinant influenza A(H3N2) viruses. The recombinant 1968 virus containing the 2017 polymerase complex (1968x2017pol) showed modest increased replication titers compared to the 1968 wildtype recombinant virus at all timepoints. Accordingly, the recombinant 2017 virus with the 1968 polymerase complex (2017x1968pol) demonstrated significantly lower replication kinetics compared to the 2017 wildtype recombinant virus, which was most pronounced at later timepoints resulted in an almost 2 log difference in replication. The recombinant 2017 virus containing the 1968HA and NA (2017x1968HANA) replicated to higher levels compared to the 2017 wildtype virus and, similarly, the recombinant 1968 virus containing the 2017HA and NA (1968x2017HANA) replicated to lower titers compared to the recombinant 1968 wildtype virus, showing a role for HA and NA in the lower replication capacity of modern viruses. The highest titer of the 1968x2017HANA recombinant virus occurred at 72 h, whereas the 1968 wildtype virus reached peak titers at 48 h. The 2017x1968HANA virus demonstrated significantly increased replication compared to the 2017 wildtype recombinant virus, which was most pronounced at 72 h. These results indicate that both the observed evolutionary increase in polymerase complex activity over time as well as changes in HA and NA over time affect the viral replication kinetics.

### HA glycan micro-array showed changes in preferential binding to glycan-moieties over time

Cultured A549, MDCK, and HAE cells have different sialic acid profiles. Our observation that both the RNA polymerase genes as well as the HA and NA genes contributed to the difference in viral titer between the 1968 and 2017 isolate suggested that differences sialic acid binding preference may contribute to the observed virus growth differences and overall viral fitness. To further investigate the discrepancies between viral replication on HAE cells and the polymerase complex activity of these influenza A(H3N2) viruses and the role of HA in decreased viral growth, we investigated the receptor binding preference of these viruses using a glycan micro-array. Human seasonal influenza A(H3N2) viruses bind to α2-6 linked sialic acid receptors (SIA), whereas avian influenza viruses favor α2-3 linked SIA. Recently it was shown that Influenza A(H3N2) viruses preferentially bind to extended N- and O-glycans terminating in α2-6 linked sialic acid, and that more recent influenza A(H3N2) viruses have evolved a preference for receptors with branched glycans with extended poly-N-acetyl-lactosamine chains (Peng et al. 2017; Broszeit et al. 2021).

**Figure 5.**
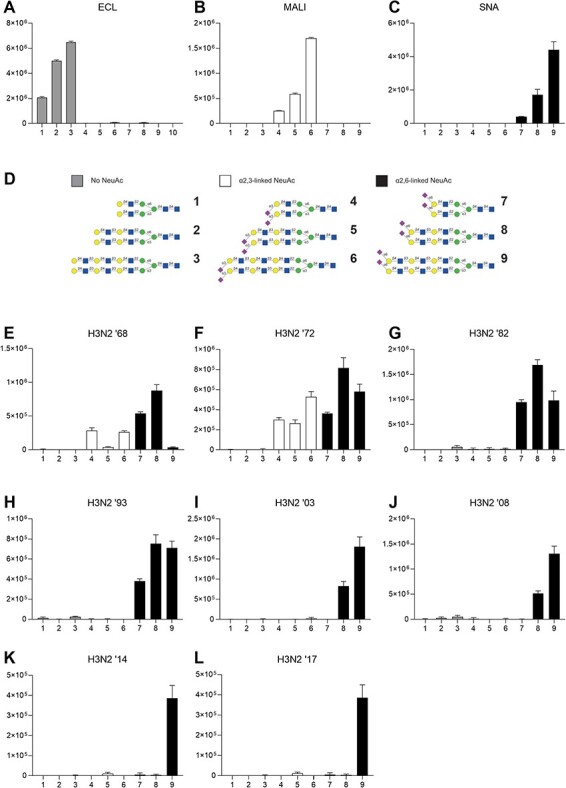
**HA receptor binding has changed over time to favor complex branched glycans**. Glycan array characterizing receptor binding specificity of selected influenza A(H3N2) virus isolates. (A) ECL, negative control, (B) MALI, positive control α2,3 linked neuraminic acid binding, (C) SNA, positive control α2,6 linked neuraminicacid binding. (D) Panel demonstrates branching of nine glycans used in glycan array assay. Differential receptor binding specificity of the selected influenza A(H3N2) viruses from 1968 (E) through 2017(L). Y-axes indicate binding in relative fluorescent units.

To investigate the glycan binding preferences of the eight influenza A(H3N2) viruses, we performed a glycan array using whole viruses. Figure 5 shows that for the influenza A(H3N2) virus from 1968 (Fig. 5E) the virus binds to shorter as well as larger branched α2-6 linked SIA but also showed residual binding to α2-3 linked SIA, as the HA was acquired through reassortment with an avian H3 virus HA. The 1972 virus (Fig. 5F) shows a similar pattern. From 1982 onward (Fig. 5G-L), however, the influenza A(H3N2) viruses show preferential α2-6 linked sialic acid binding, having lost the capacity to bind avian-type α2-3 linked sialic acids. Both the 2003 and 2008 influenza A(H3N2) viruses (Fig. 5I-J) show a more restricted pattern of binding preferentially to more complex branched α2-6 linked sialic acids, having lost the capacity to bind shorter branches with α2-6 linked SIA. Both the 2014 and 2017 viruses (Fig. 5K-L) demonstrate binding solely to long branched complex α2-6 linked sialic acids and thereby exhibit more restricted receptor binding. These results demonstrate that the HA binding or avidity changed for the H3N2 viruses analyzed.

## Discussion

Seasonal influenza A(H3N2) viruses have continued to circulate in the human population for over 50 years since their pandemic introduction in 1968. Our phylogenetic analysis confirms earlier studies (Nelson et al. 2006; Rambaut et al. 2008; Westgeest et al. 2014), showing that there is ongoing evolution in the influenza A(H3N2) virus polymerase complex gene segments, although the rate of nucleotide substitution is higher for the HA and NA gene segments compared to the internal genes.

To study the evolution of the influenza A(H3N2) virus polymerase complex at the phenotype level, we selected eight viruses spanning nearly 50 years of influenza virus evolution. The viruses we selected were representative for influenza A(H3N2) viruses circulating during their corresponding season based on our phylogenetic analysis.

Our data clearly show that there is a gradual increase in polymerase complex activity from 1968 to 2017, without affecting the balance between replication and transcription. These relative differences in polymerase complex activity between the viruses were more pronounced at 33°C and most apparent early during the transcription process. When we reassorted the polymerase complex gene segments of the 1968 virus with those of the 1972, 1982, or 2017 virus and quantified their polymerase complex activity in mini-genome assays, we consistently found that the PB1 and NP gene segments contributed most to the increase in polymerase complex activity over time. These results indicate that during the evolution of the polymerase complex selection pressure acts on multiple polymerase complex subunits, which are highly dependent on each other for polymerase complex function.

We have identified numerous amino-acid substitutions within the influenza A(H3N2) virus polymerase complex that became fixed over time, but do not understand their effect on polymerase complex function for most of these. A more comprehensive functional analysis of individual substitutions within the influenza A(H3N2) virus polymerase complex genes or a step-wise introduction of these amino-acid changes in influenza A(H3N2) viruses from 1968 onward could elucidate which of these substitutions affect polymerase complex function, alone or in combination.

The polymerase complex of the 2003 influenza A(H3N2) virus demonstrated low transcriptional polymerase complex activity in HEK293T cells in mini-genome assays and in virus inoculated MDCK reporter cells, whereas in A549 cells, the polymerase complex activity of this virus was high in mini-genome assays. Given the differences in cellular origin of these cell lines, it is tempting to speculate that the polymerase complex of the 2003 influenza A(H3N2) virus exhibits some tissue tropism or restriction, but it requires further study to elucidate the underlying mechanism.

Surprisingly, our results indicate that viruses with a relatively low polymerase complex activity in mini-genome assays and replication in MDCK cells such as the 1968, 1972, and 1982 viruses replicated faster in primary respiratory epithelial cells than the viruses with higher polymerase complex activity in mini-genome assays such as the 1993, 2008, and 2014 viruses. Replication experiments on primary HAE cells using reassorted viruses clearly demonstrate the positive contribution of the polymerase complex of more recent A(H3N2) viruses as well as the negative effect of HA and NA on replication kinetics. The polymerase complex of 2017 influenza A(H3N2) virus positively affected replication, while the 2017HA and NA gene segments negatively influenced replication.

To reconcile the discrepancy between the polymerase complex activity of these influenza A(H3N2) viruses and their replication on HAE cells, we hypothesize that the increase of influenza A(H3N2) virus polymerase complex activity is compensatory for a loss of viral fitness caused by the effects of antigenic driven evolution of HA and NA. Since the polymerase complex activity of influenza A(H3N2) viruses has increased gradually since 1968, the selection pressures driving the polymerase complex evolution must also evolve to exert a form of continuous selection pressure, most likely by immune pressure. For HA, this resulted in multiple antigenic cluster transitions and increased glycosylation (Altman et al., 2019; Koel et al. 2013). However, these processes likely also come with a fitness cost in terms of receptor binding avidity and specificity, an increase in cellular processing time of HA through the endoplasmic reticulum (ER) (Gallagher et al. 1992; Roberts, Garten, and Klenk 1993) or induction of ER stress pathways (Hrincius et al. 2015) due to changes in HA glycosylation. Interestingly, many of the mutations in the HA globular head domain which facilitate antigenic cluster transitions, as well as glycosylation sites involved in glycan shielding, are close to the receptor binding site (RBS), which illustrates the relationship between antigenic evolution and receptor binding specificity. We show here that the preferred binding of glycan moieties evolved over time to become more restricted, confirming earlier studies (Yang et al. 2015; Peng et al. 2017). For HA, changes in glycan binding preference could lower viral replication through less promiscuous usage of receptors on the cell surface, since earlier viruses bind a wider variety of receptors than later viruses, possibly explaining the decreased replication as observed in the HAE experiments.

A possible compensatory role for the polymerase complex for the decrease in viral fitness might have to do with loss of function of HA or post-translational modification of HA. Due to increased glycosylation of HA, delayed processing of HA in the ER and subsequent compartments due to complex enzymatic modification of glycan moieties can result in reduced expression of HA on the cellular membrane. A compensatory increase in mRNA due to increased polymerase complex activity could overcome this loss of HA processing, favoring viruses which exhibit higher polymerase complex activity. On the other hand, for both influenza A(H2N2) (Tsuchiya et al. 2002) and A(H3N2) viruses (Abe et al. 2004) it has been suggested that the expression of HA on the cell membrane is not affected by the number of glycan moieties on HA accumulated over time when the HA of these viruses where cloned into expression vectors. However, these experiments did not involve the influenza virus polymerase complex or other gene segments which could contribute to the regulation of transcription, ER processing, and expression of HA on the cell membrane. Due to continuous antigenic pressure, HA has accumulated numerous substitutions in and around the receptor binding site of HA. This is very likely the driving force of the higher receptor specificity. A less promiscuous receptor binding might be unbeneficial for viral fitness and therefore polymerase activity could possibly help compensate for this fitness loss.

In addition to the compensatory role for evolution of the polymerase complex described above, the polymerase complex might also be subject to T-cell mediated immune pressure, adding to the genetic diversity of the polymerase genes and selection of specific amino acid changes (Rimmelzwaan et al. 2009).

In this study, we demonstrated that the evolution of the influenza A(H3N2) virus has resulted in an increase in polymerase complex activity over time, which is associated with HA and NA evolution, largely due to immunological selection pressures on HA and NA. However, the polymerase complex activity as measured in mini-genome assays reflects mostly the transcriptional activity of the complex under strict laboratory conditions and does not quantify other functions of the polymerase complex such as vRNA and cRNA synthesis nor the dynamics between the various RNA species during natural infection. qPCR analysis showed a preserved balance between mRNA, vRNA, and cRNA at 24 h after transfection; yet, the temporal dynamics of these RNA species over the course of influenza virus infection remain to be elucidated. Other functions of the polymerase complex, such as replication fidelity and the generation of small viral RNAs which may induce innate immune responses, have not been studied here. Future research focusing on various aspects of polymerase complex activity and a possible compensatory role for the loss of HA functionality is needed to fully understand the evolution of the influenza A(H3N2) polymerase complex, including similar studies in H1N1 and H1N1pdm2009 viruses.

## Supplementary Material

veae030_Supp
