# Supplementary material for: Long-term evolution of human seasonal influenza virus A(H3N2) is associated with an increase in polymerase complex activity
Source: Virus Evol. 2024 May 4;10(1):veae030. doi: 10.1093/ve/veae030 (PMC11131032; doi:10.1093/ve/veae030)
Supplement: veae030_Supp [file veae030_supp.zip › Vigeveno et al H3N2 polymerase complex_Supplemental figure 1_v2.pdf]

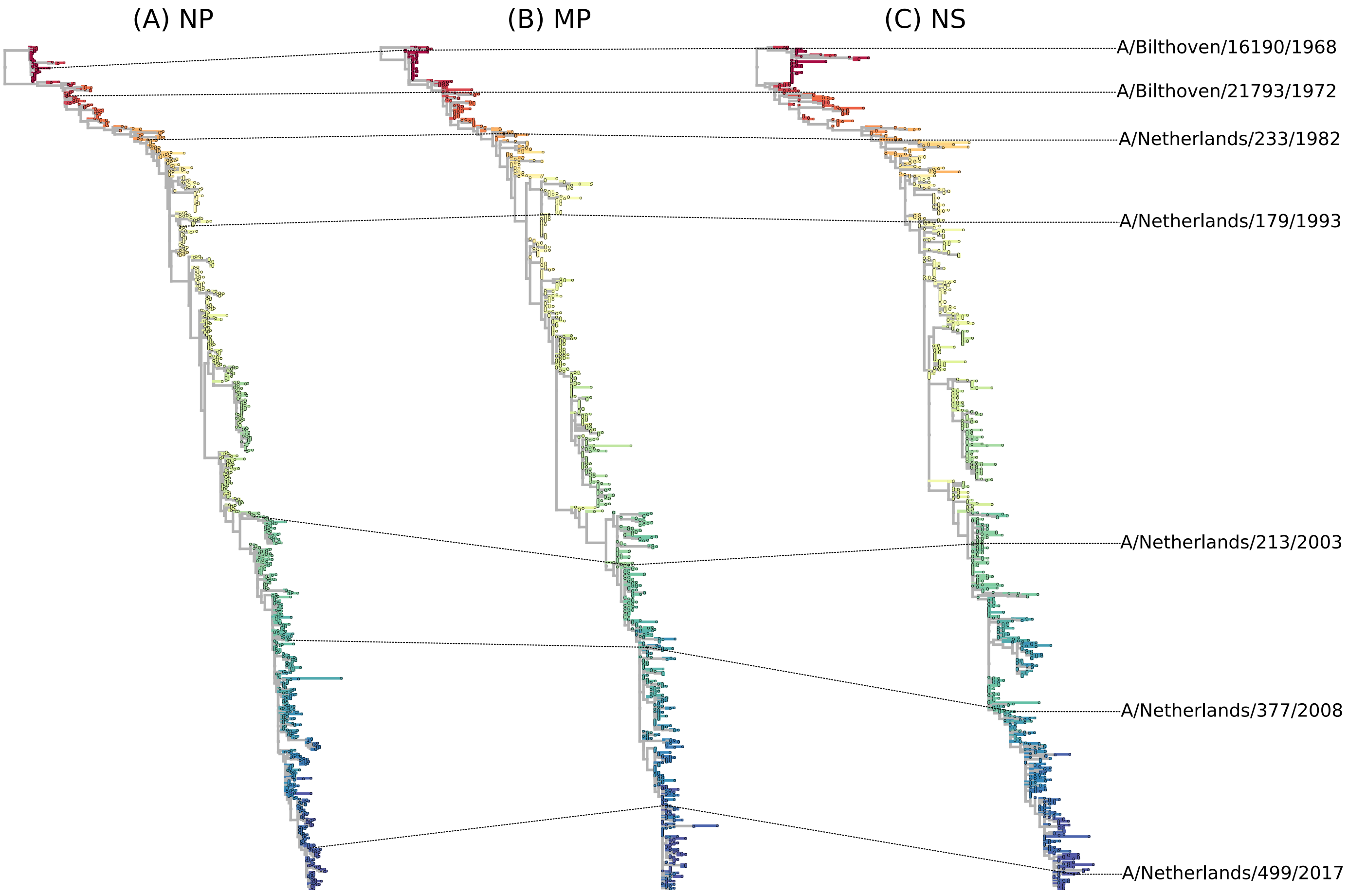

Supplemental figure 1. Phylogenetic analysis demonstrate ongoing genetic evolution of human influenza A(H3N2) virus NS, NP and M gene segments between 1968 and 2017.

Phylogenetic trees of influenza A(H3N2) gene segments NS (A) NP (B) and M (C) demonstrate ongoing genetic evolution from 1968 onward. Trees are color-coded to antigenic evolution based on antigenic mapping (5, 8). Influenza A(H3N2) viruses used for subsequent phenotypic characterization are annotated in each phylogenetic tree.
