# Supplementary material for: Long-term evolution of human seasonal influenza virus A(H3N2) is associated with an increase in polymerase complex activity
Source: Virus Evol. 2024 May 4;10(1):veae030. doi: 10.1093/ve/veae030 (PMC11131032; doi:10.1093/ve/veae030)
Supplement: veae030_Supp [file veae030_supp.zip › Vigeveno et al H3N2 polymerase complex_Supplemental figure 2.pdf]

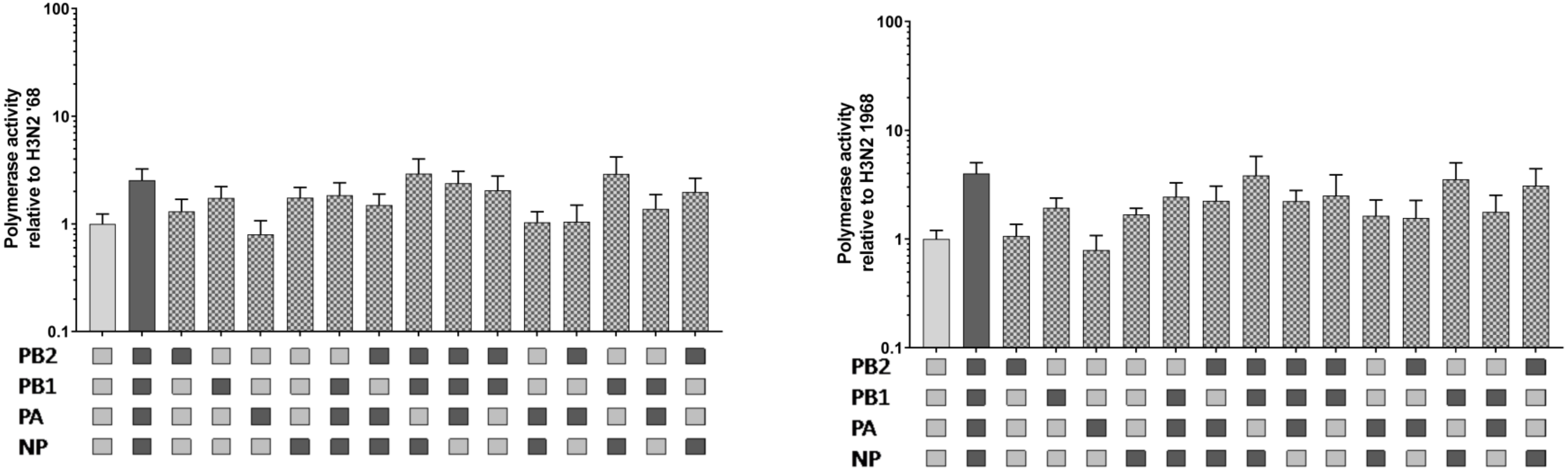

Supplemental figure 2. Increased polymerase complex activity in influenza A(H3N2) viruses from 1968 to 2017 attributed to PB1 and NP gene segments. Polymerase complex activity of influenza A(H3N2) viruses from (A) 1968 and 1972 and reassortants thereof (B) 1968 and 1982 and reassortants thereof in HEK293T cells incubated at 37°C as measured in mini-genome assays.
