# Supplementary material for: Long-term evolution of human seasonal influenza virus A(H3N2) is associated with an increase in polymerase complex activity
Source: Virus Evol. 2024 May 4;10(1):veae030. doi: 10.1093/ve/veae030 (PMC11131032; doi:10.1093/ve/veae030)
Supplement: veae030_Supp [file veae030_supp.zip › Vigeveno et al H3N2 polymerase complex_Supplemental table 1.docx]

Supplemental table 1. Amino-acid changes in the PB2, PB1, PA and NP gene segments of eight influenza A(H3N2) viruses used to characterize the evolution of the seasonal influenza A(H3N2) polymerase complex.

The pandemic influenza A(H3N2) virus was used as the reference. Amino-acid changes were classified as (A) fixed, (B) multiple amino-acid changes at one position, or (C) unique to one of the influenza A(H3N2) viruses after 1968. Amino-acid positions which did not change in one of the seven influenza A(H3N2) viruses compared to the 1968 influenza A(H3N2) virus are not listed.

A. Amino-acid changes which fixated over time relative to the 1968 influenza A(H3N2) virus, listed chronologically in PB2, PB1, PA and NP respectively.

| **Amino-acid position** | **Influenza A(H3N2) virus** | | | | | | | |
| --- | --- | --- | --- | --- | --- | --- | --- | --- |
|  | **1968 (reference)** | **1972** | **1982** | **1993** | **2003** | **2008** | **2014** | **2017** |
| PB2_105 | M | V | V | V | V | V | V | V |
| PB2_120 | E | D | D | D | D | D | D | D |
| PB2_344 | L | V | V | V | V | V | V | V |
| PB2_394 | V | I | I | I | I | I | I | I |
| PB2_495 | I | V | V | V | V | V | V | V |
| PB2_526 | K | R | R | R | R | R | R | R |
| PB2_682 | G | S | S | S | S | S | S | S |
| PB1_118 | X | R | R | R | R | R | R | R |
| PB1_336 | V | I | I | I | I | I | I | I |
| PB1_361 | S | R | R | R | R | R | R | R |
| PB1_395 | I | L | L | L | L | L | L | L |
| PB1_430 | R | K | K | K | K | K | K | K |
| PA_241 | Y | C | C | C | C | C | C | C |
| PA_269 | R | K | K | K | K | K | K | K |
| PA_387 | V | I | I | I | I | I | I | I |
| PA_630 | D | E | E | E | E | E | E | E |
| PA_684 | E | G | G | G | G | G | G | G |
| NP_343 | V | L | L | L | L | L | L | L |
| NP_423 | P | S | S | S | S | S | S | S |
| NP_456 | M | V | V | V | V | V | V | V |
| NP_459 | Q | R | R | R | R | R | R | R |
| PB2_107 | S | S | N | N | S | S | S | S |
| PB2_456 | N | N | S | S | S | S | S | S |
| PB1_121 | R | R | K | K | K | K | K | K |
| PB1_486 | R | R | K | K | K | K | K | K |
| PB1_584 | R | R | Q | Q | Q | Q | Q | Q |
| PA_204 | R | R | K | K | K | K | K | K |
| PA_332 | P | P | S | S | S | S | S | S |
| NP_186 | V | V | I | I | I | I | I | I |
| NP_217 | G | G | S | S | S | S | S | S |
| NP_286 | A | A | S | S | S | S | S | S |
| NP_334 | N | N | H | H | H | H | H | H |
| NP_411 | A | A | T | T | T | T | T | T |
| NP_421 | D | D | E | E | E | E | E | E |
| PB2_194 | Q | Q | Q | R | R | R | R | R |
| PB2_338 | I | I | I | V | V | V | V | V |
| PB2_569 | T | T | T | A | A | A | A | A |
| PB1_216 | S | S | S | G | G | G | G | G |
| PB1_581 | E | E | E | D | D | D | D | D |
| PB1_621 | Q | Q | Q | R | R | R | R | R |
| PA_277 | F | F | F | Y | Y | Y | Y | Y |
| PA_311 | M | M | M | I | I | I | I | I |
| PA_618 | T | T | T | A | A | A | A | A |
| PA_716 | R | R | R | K | K | K | K | K |
| PB2_340 | R | R | R | R | K | K | K | K |
| PB2_590 | G | G | G | G | S | S | S | S |
| PB2_697 | L | L | L | L | I | I | I | I |
| PB1_586 | K | K | K | K | R | R | R | R |
| PB1_619 | D | D | D | D | N | N | N | N |
| PB1_709 | V | V | V | V | I | I | I | I |
| PA_312 | R | R | R | R | K | K | K | K |
| PA_343 | A | A | A | A | S | S | S | S |
| PA_382 | D | D | D | D | E | E | E | E |
| PA_557 | M | M | M | M | I | I | I | I |
| PA_573 | I | I | I | I | V | V | V | V |
| NP_17 | E | E | E | E | D | D | D | D |
| NP_65 | R | R | R | R | K | K | K | K |
| NP_98 | K | K | K | K | R | R | R | R |
| NP_127 | D | D | D | D | E | E | E | E |
| NP_136 | M | M | M | M | I | I | I | I |
| NP_146 | T | T | T | T | A | A | A | A |
| NP_197 | I | I | I | I | V | V | V | V |
| NP_375 | E | E | E | E | G | G | G | G |
| NP_384 | R | R | R | R | G | G | G | G |
| NP_406 | I | I | I | I | T | T | T | T |
| PB2_249 | E | E | E | E | E | G | G | G |
| PB2_451 | I | I | I | I | I | V | V | V |
| PB1_52 | K | K | K | K | K | R | R | R |
| PB1_113 | V | V | V | V | V | A | A | A |
| PB1_576 | L | L | L | L | L | I | I | I |
| PA_101 | E | E | E | E | E | G | G | G |
| NP_52 | Y | Y | Y | Y | Y | H | H | H |
| NP_131 | A | A | A | A | A | S | S | S |
| NP_239 | M | M | M | M | M | V | V | V |
| NP_280 | V | V | V | V | V | A | A | A |
| NP_312 | V | V | V | V | V | I | I | I |
| PB2_353 | K | K | K | K | K | K | R | R |
| PB2_588 | I | I | I | I | I | I | T | T |
| PB2_613 | T | T | T | T | T | T | A | A |
| PB1_587 | A | A | A | A | A | A | T | T |
| PA_396 | D | D | D | D | D | D | E | E |
| PA_409 | N | N | N | N | N | N | S | S |
| PA_668 | V | V | V | V | V | V | I | I |
| PA_675 | N | N | N | N | N | N | K | K |
| PB2_299 | R | R | R | R | R | R | R | K |

B. Multiple amino-acid changes at one position

| **Amino-acid position** | **Influenza A(H3N2) virus** | | | | | | | |
| --- | --- | --- | --- | --- | --- | --- | --- | --- |
|  | **1968**  **(reference)** | **1972** | **1982** | **1993** | **2003** | **2008** | **2014** | **2017** |
| PB2_461 | I | V | V | V | V | I | V | V |
| PB2_559 | I | T | T | T | A | A | A | A |
| PB1_179 | M | M | I | I | M | M | M | M |
| PB1_741 | V | A | S | S | S | S | S | S |
| PA_208 | T | T | T | S | S | T | T | T |
| PA_256 | K | R | R | R | Q | Q | Q | Q |
| PA_272 | D | D | D | N | N | N | S | S |
| PA_421 | T | I | I | I | I | V | V | V |
| NP_77 | K | R | R | R | K | K | K | K |
| NP_103 | K | K | R | R | K | K | K | K |
| NP_259 | L | L | S | S | L | L | L | L |
| NP_373 | A | T | N | N | N | N | N | N |
| NP_425 | I | I | V | V | I | I | I | I |
| NP_473 | A | T | T | T | T | T | T | A |

C. Amino-acid mutations unique to one of the influenza A(H3N2) viruses after 1968

| **Amino-acid position** | **Influenza A(H3N2) virus** | | | | | | | |
| --- | --- | --- | --- | --- | --- | --- | --- | --- |
|  | **1968**  **(reference)** | **1972** | **1982** | **1993** | **2003** | **2008** | **2014** | **2017** |
| PB2_356 | V | V | V | V | **I** | V | V | V |
| PB2_553 | I | I | I | **V** | I | I | I | I |
| PB2_560 | V | V | V | V | V | **I** | V | V |
| PB1_292 | N | N | N | N | N | N | **S** | N |
| PB1_375 | S | S | S | S | **N** | S | S | S |
| PB1_745 | K | K | K | K | K | **R** | K | K |
| PA_216 | D | D | D | D | D | D | D | **S** |
| PA_243 | E | E | E | E | E | E | **G** | E |
| PA_321 | Y | Y | Y | Y | Y | Y | **N** | Y |
| PA_325 | P | P | **S** | P | P | P | P | P |
| PA_350 | N | N | N | **T** | N | N | N | N |
| NP_351 | K | K | K | K | K | **R** | K | K |
