# Supplementary material for: Long-term evolution of human seasonal influenza virus A(H3N2) is associated with an increase in polymerase complex activity
Source: Virus Evol. 2024 May 4;10(1):veae030. doi: 10.1093/ve/veae030 (PMC11131032; doi:10.1093/ve/veae030)
Supplement: veae030_Supp [file veae030_supp.zip › Supplemental Figure legends.docx]

**Supplemental Figure legends**

**Supplemental figure 1. Phylogenetic analyses demonstrate ongoing genetic evolution of human influenza A(H3N2) virus NS, NP and M gene segments between 1968 and 2017.**

Phylogenetic trees of influenza A(H3N2) gene segments NS (A) NP (B) and M (C) demonstrate ongoing genetic evolution from 1968 onward. Trees are color-coded to antigenic evolution based on antigenic mapping (5, 8). Influenza A(H3N2) viruses used for subsequent phenotypic characterization are annotated in each phylogenetic tree.

**Supplemental figure 2. Increased polymerase complex activity in human influenza A(H3N2) viruses from 1968 to 2017 attributed to PB1 and NP gene segments**.

Polymerase complex activity of influenza A(H3N2) viruses from (A) 1968 and 1972 and reassortants thereof (B) 1968 and 1982 and reassortants thereof in HEK293T cells incubated at 37°C as measured in mini-genome assays.
